# In Vitro Evaluation of Extracts From Ixora Species for a Potential Phytosomal Formulation

**DOI:** 10.7759/cureus.55396

**Published:** 2024-03-02

**Authors:** Jasmin Sajini Rajayan, Vinodhini Chandrasekar, Chamundeeswari Duraipandian, Karthik Rajendran

**Affiliations:** 1 Pharmaceutical Chemistry, Sri Ramachandra Institute of Higher Education and Research, Chennai, IND; 2 Pharmacy, Sri Ramachandra Institute of Higher Education and Research, Chennai, IND; 3 Pharmacy, Meenakshi Academy of Higher Education and Research, Chennai, IND; 4 Bioanalytics and Analytics, Scitus Pharma Services Private Limited, Chennai, IND

**Keywords:** flower extract, leaf extract, in vitro skin anticancer study, phytosome formulation, quercetin, flavonoids, ixora alba, ixora coccinea

## Abstract

Background

*Ixora* species are perennial shrubs and flowering plants belonging to the family *Rubiaceae*. The leaf and flower parts of *Ixora coccinea *(*I. coccinea*)* *and*Ixora alba *(*I. alba*)* were* aimed at isolating their active fractions. The present study was to determine in vitro antitumor activity against malignant melanoma cell lines for phytosome formulation.

Materials and methods

Two species, *I. coccinea* (red flowers and leaves) and *I. alba* (white flowers and leaves), were selected, and this study focused on determining the active fraction by comparing the in vitro antimicrobial and antioxidant potentials of petroleum ether, chloroform, ethyl acetate, and hydroalcoholic (ethanol:water, 70:30 v/v) extracts. The identified potent extract was subjected to in vitro anticancer activity in malignant melanoma cell lines.

Results

A phytochemical study revealed phytosterols, flavonoids, proteins, amino acids, alkaloids, carbohydrates, phenols, tannins, and diterpenes. The 2,2-diphenyl-1-picrylhydrazyl (DPPH) assay was used to evaluate the antioxidant effect of *I. coccinea and I. alba* leaf and flower extracts. In the DPPH assay, *I. coccinea *flower hydroalcoholic extract (ICFHA) had an IC_50_ value of 248.99 µg/mL, and *I. coccinea *leaf hydroalcoholic extract (ICLHA) had an IC_50_ value of 268.87 µg/mL. These two extracts had a lower value with a higher antioxidant effect. In the total antioxidant assay, *I. coccinea* leaf ethyl acetate extract (ICLEA) and *I. coccinea *leaf chloroform extract (ICLCE) have 77.4 ± 0.05 and 68.9 ± 0.03 mg of ascorbic acid equivalent per gm of extract, respectively. These two extracts exhibited a high antioxidant effect. The antimicrobial potential was evaluated using selected bacterial and fungal strains using the agar-well diffusion method. Petroleum ether and chloroform extracts of *I. coccinea* and *I. alba* leaves and flowers did not possess antimicrobial activity with any of the bacterial or fungal strains. An ethyl acetate extract and a hydroalcoholic extract of *I. coccinea* leaves and flowers showed antimicrobial activity against *Enterococcus faecalis*, *Candida albicans*, and *Staphylococcus aureus*. An ethyl acetate extract of *I. coccinea* flower and a hydroalcoholic extract of *I. alba* leaf showed a significant zone of inhibition when compared with standard chloramphenicol for all three selected strains, which may be due to the presence of active phytoconstituents. ICLHA showed a MIC of ≤300 µg/mL for *Enterococcus faecalis* and *Staphylococcus aureus *and ≤400 µg/mL for *Candida albicans* microbial strains. The high total flavonoid content was reported in ICLEA at 771.31 µg/mL and in *I. coccinea* flower ethyl acetate extract (ICFEA) at 694.69 µg/mL. High-performance thin layer chromatography (HPTLC) analysis showed a high quercetin (QCE) content in the ICLEA extract. To prove the in vitro skin anticancer activity, an MTT assay was performed for the ICLEA extract in a malignant melanoma cell line, and the IC50 value was reported as 7.96 µg/mL.

Conclusion

*I. coccinea* leaf ethyl acetate extract revealed a significant total flavonoid content in analysis through the aluminum chloride method, and the presence of a high QCE content was confirmed by HPTLC analysis. The in vitro skin anticancer activity of ICLEA was confirmed by the MTT assay; therefore, it was concluded that the ICLEA extract was a potent fraction and was selected to develop a phytosome.

## Introduction

Herbal medicine plays a significant role as an alternative to synthetic medication due to its reduced cost and lack of negative side effects. Plants have been used to separate various chemicals. Traditional medicine is practiced in many countries [1]. A family of flowering plants called *Rubiaceae* includes the genus *Ixora*. There were approximately 500 species of trees and shrubs. Some of the common names of *Ixora* are West Indian Jasmine, Rangan, Kheme, Ponna, Chann Tanea, Techi, Pan, Santan, Jarum-Jarum, Jungle Flame, and Jungle Geranium. Although more species were available, only a few *Ixora* species were cultivated, and their pharmacological effects were investigated [2]. The flower portions of plants that produce yellow, red, or blue coloration include flavonoids, which are well known for their antioxidant qualities and are responsible for protecting them from attack by microbes and insects. Compared with other active plant compounds, they have low toxicity [3]. Plant phenolics have several actions, including protection against pathogens, UV ray protection, and pigmentation [4].

There have been reports of quercetin (QCE), rutin, kaempferol-3-rutinoside, leucocyanidin glycoside, d-mannitol, and ursolic acid in the blooms of *Ixora coccinea* (*I. coccinea*). The blooms of *I. coccinea* also contain anthocyanins [5]. The heated decoction from shade-dried flowers and coconut oil is externally used to treat eczema. According to previous studies, the flower’s hydro-methanolic extract (70%) exhibits significant in vitro free radical scavenging action [6]. In mice, an aqueous leaf extract from *I. coccinea *showed antinociceptive activity [7]. Triterpenoid and ursolic acid are found in the flower extract of* I. coccinea* [8]. Researchers have discovered that the known alkaloid camptothecin is primarily responsible for the anticancer activity of the leaves of *I. coccinea* (*Rubiaceae*) [9]. Flowers from *I. coccinea* constitute a component of an ayurvedic formulation used to treat oral cancer [10]. *Ixora alba* (*I. alba*), a small- to medium-sized hardy shrub in the *Rubiaceae* family, is grown for ornamental purposes. *Ixora* species have been employed in traditional Indian medical systems for the treatment of ulcers, diarrhea, and, more recently, antitumor activity [11].

## Materials and methods

Plant authentication

Selected *Ixora *species were authenticated by Prof. P. Jayaraman, PhD, Institute of Herbal Botany, Chennai: *I. coccinea *specimen certificate no.: PARC/2019/4016 and* I. alba* specimen certificate no.: PARC/2019/4017.

Extraction of plant material

The selected *Ixora *species, *I. coccinea* and *I. alba*, were collected in February and thoroughly washed with distilled water before being shadow-dried and used for further extraction. The plant materials were coarsely powdered and weighed to ensure complete extraction. Petroleum ether, chloroform, ethyl acetate, and hydroalcoholic (ethanol:water, 70:30, v/v) solvents were used for extraction. The maceration method was chosen to reduce the cost of evaporation and minimize heat damage to the thermolabile constituents. The plant materials were macerated for 72 hours, followed by 48 and 24 hours of maceration with occasional shaking. The extracts were filtered using Whatman filter paper, and the solvents were distilled, dried, stored in a container, and kept in a desiccator. The weight of the extracts was recorded. The yield was expressed as % w/w [12].

Elemental analysis

The instrument used for elemental analysis is inductively coupled plasma mass spectrometry (ICP-MS) [13]. The elements detected were arsenic (As), cadmium (Cd), lead (Pb), mercury (Hg), and iron (Fe).

Phytochemical analysis

Using various reagents, extracts of *I. coccinea* and *I. alba *were phytochemically screened for the presence of alkaloids, anthraquinone cardiac glycosides, coumarin flavonoid saponins, tannins, saponins, proteins, amino acids, and terpenoids [14].

Antioxidant studies

2,2-Diphenyl-1-Picrylhydrazyl (DPPH) Assay

To conduct the radical scavenging assay, DPPH was purchased from Merck, Mumbai, India. It was prepared using methanol-based dissolution. Plant extract stock solutions (50 µg) from the stock series of 50 to 1000 µg were made. It was incubated for 30 minutes with 0.1 mL of extract and 1.9 mL of DPPH, and the absorbance of the reaction mixture was measured at 517 nm using a UV spectrophotometer. The formula was used to compute free radical scavenging [15].

% Inhibition = (OD of control- (OD of the test) / OD of control)) × 100

where the OD of the control is the absorbance of the negative control and the OD of the test is the absorbance of the sample.

Total Antioxidant Assay

A 1000 µg/mL stock solution (plant extract in dimethylsulfoxide (DMSO)) was prepared and diluted to concentrations ranging from 50 µg/mL to 1000 µg/mL. Ascorbic acid was used as a control. One milliliter of the reagent solution (0.6 M sulphuric sulfuric, 28 mM sodium phosphate, and 4 mM ammonium molybdate) was mixed with 0.1 mL of the sample solution, and the absorbance at 695 nm against the blank was measured using a UV-visible spectrophotometer. The antioxidant activity was measured in grams per milliliter of ascorbic acid equivalents (AAE) [16].

Antimicrobial activity using the agar-well diffusion method

To evaluate the antimicrobial activity of* I. coccinea* and *I. alba*, the concentrations of the samples used were 250 µg, 500 µg, and 1000 µg. DMSO was used as the control, and chloramphenicol (50 µg) was used as the standard. The medium used was Mueller-Hinton Media. Gram-positive strains of *Staphylococcus aureus* (ATCC-25932), *Enterococcus faecalis* (ATCC-29212), and *Streptococcus pyogenes* were used. Gram-negative strains of *Escherichia coli* (ATCC-25922), *Klebsiella pneumoniae* (ATCC-13883), and *Pseudomonas aeruginosa* (ATCC-27853) were used. Fungal strains of *Candida albicans* (ATCC-10231) and *Cryptococcus neoformans *(ATCC-14116) were used. Using a sterile cotton swab, bacterial solutions were evenly distributed over sterile agar Petri dishes. The plates were then held for five minutes. Then, 7-mm-diameter wells in each of the agar Petri dishes were punched, and each plate contained 30 μL of petroleum ether, chloroform, ethyl acetate, and hydroalcoholic extracts of the leaves and flowers of *I. coccinea* and* I. alba*. The plates were then left to diffuse at room temperature for one hour. Subsequently, the plates were incubated for 24 hours at 37°C. Following incubation, confluent bacterial growth was noticed, and the scale was used to quantify the length in millimeters of the bacterial growth inhibition zone. As a benchmark, chloramphenicol (50 μg/disc) was used. Every experiment was conducted in duplicate, and the averages were taken as the result [17].

Minimum inhibitory concentration (MIC)

The MIC was determined by the broth dilution method [18]. In a sterile test tube, 2 mL of brain heart infusion broth was added, and each 2 mL sample of 300 µg/mL, 400 µg/mL, and 500 µg/mL was mixed separately and diluted to 100 mL of organism suspension. The samples were incubated at 37°C for 24 hours. *Staphylococcus aureus*, *Enterococcus faecalis*, and *Candida albicans* were used in this study.

Total flavonoid content

The extracted sample in methanol was produced at 10 mg/mL. By diluting standard QCE (25-1000 µg/mL) in methanol, a calibration curve was created. Six hundred milliliters of methanol were used to dilute 200 µL of the QCE extract before adding 2.0 mL to 40 µL of a 10% (w/v) aluminum chloride solution and 40 µL of a 1 M potassium acetate solution. The combination was given 30 minutes to rest at room temperature. Subsequently, a UV-VIS spectrophotometer was used to quantify the mixture’s peak absorbance at 415 nm [19].

High-performance thin layer chromatography (HPTLC)

HPTLC was performed on 20-cm silica gel 60 HPTLC plates, which were dried at 110°C for 30 minutes before use. The silica gel-coated plate was used for all standards. Toluene, ethyl acetate, formic acid, and methanol (5.5:3:1:0.5 v/v/v/v) were used to develop plates in a horizontal or saturated twin-trough developing chamber. The retention factor was computed using the following formula:

Retention factor = distance moved by the sample/ distance moved by the solvent

UV light is the most commonly used non-destructive visualization method for thin layer chromatography (TLC) plates. The TLC Visualizer was used to detect the plates containing standards and samples under white light illumination, long-wavelength UV light at 366 nm, and short-wavelength UV light at 254 nm.

The analyses were performed on 10 × 10 mm glass plates using a Linomat 5 (CAMAG, Muttenz, Switzerland) automated spray system. Based on the results obtained from the DPPH assay, total antioxidant, total flavonoid, and antimicrobial activity, the following *I. coccinea* leaf ethyl acetate (ICLEA), *I. coccinea* flower ethyl acetate (ICFEA), *I. alba* leaf chloroform (IALC), and *I. alba* flower ethyl acetate (IAFEA) extracts were selected for HPTLC analysis using QCE as the standard. All samples were applied with a 5 µl sample syringe. Five µL samples were used as 5-mm bands with a 10-mm distance between the band centers. Plates containing samples and the standard (QCE) were developed for 20 minutes in a saturated vertical glass chamber with a mobile phase. The migration distance of the mobile phase was calculated. The plates were dried and visualized in the TLC visualizer at various wavelengths after development.

QCE was quantified using an imaging processing method based on HPTLC. In brief, standard QCE concentrations of 10, 50, 100, 250, 500, and 1000 µg/mL were used. The sample was then taken at a concentration of 10 mg/ml and plotted alongside the standard in a silica-precoated TLC plate. The plate was then run through the same solvent system used in the previous standardized study (toluene:ethyl acetate:formic acid:methanol (5.5:3:1:0.5, v/v/v/v)) and sprayed with an ethanolic aluminum chloride solution to improve the visualization of the flavonoid-based sample spots. Finally, the derivatized (sprayed) plate was scanned using HPTLC image software for spot quantification [20].

In vitro anticancer evaluation using the MTT assay

The National Centre for Cell Science in Pune, India, donated human A375 cell lines (malignant melanoma cell lines), which were cultured in Eagle’s minimum essential medium. All cell cultures were maintained at 37°C with 100% relative humidity, 5% CO_2_^,^ and 95% air. Twice a week, the dose was altered. Using trypsin and ethylene diamine tetraacetic acid, single suspensions were prepared. By diluting the suspension with 5% FBS, 1 × 105 cells/mL of cell suspension was obtained. In 96-well plates, 100 mL (10,000 cells per well) were planted. At 37°C, it was incubated. Different concentrations of 100, 50, 25, 12.5, 6.25, 3.125, and 1.5 µg/mL extract were used to treat the cells. After drug addition, the plates were incubated at 37 °C. As a control, a medium without samples was used.

Each well received 20 µL of MTT (5 mg/mL) in phosphate-buffered saline after 48 hours of incubation, followed by a four-hour incubation period at 37°C. The MTT-containing media was turned off, and formazan crystals were dissolved in 100 µL of DMSO. At 570 nm, the absorbance was measured using a microplate reader. The following formula was used to calculate the percentage of cell inhibition:

% cell inhibition was calculated as [100 − Abs (sample) / Abs (control)] × 100.

The same methodology was used to examine the impact of ICLEA on keratinocyte cell lines (HaCaT cell line) and human dermal fibroblasts under comparable circumstances [21].

Selection of the active fraction of *Ixora* species from the preliminary analysis for phytosomal formulation

*I. coccinea* and* I. alba* leaves and flower parts from the *Ixora* species were selected for the study. Sixteen extracts were prepared using various solvents, including petroleum ether, chloroform, ethyl acetate, and hydroalcoholics. The active fraction was identified by performing preliminary analyses such as phytochemical analysis, DPPH assay, total antioxidant assay, antimicrobial assay, total flavonoid content, and HPTLC analysis. Phytochemical analysis and antioxidant studies of the DPPH assay, the total antioxidant assay, and the antimicrobial assay were performed for all 16 extracts. Of the 16 extracts based on phytoconstituents, antibacterial effect, and free radical scavenging effect, six extracts (ICLEA, ICFEA, ICFHA, IALCE, IALHA, and IAFEA) were selected. The total flavonoid contents of these six selected extracts were calculated. From the flavonoid content comparison, only four extracts (ICLEA, ICFEA, IALCE, and IAFEA) were analyzed by HPTLC and quantified for QCE content. Finally, the ICLEA extract was selected and used for the phytosomal formulation.

## Results

Extraction of plant material

The extractive value was calculated and reported as % w/w (Table 1).

**Table 1:** The extractive yield of Ixora coccinea and Ixora alba leaf and flower ICLPE: *Ixora coccinea* leaf petroleum ether; ICLCE: *Ixora coccinea* leaf chloroform extract; ICLEA: *Ixora coccinea* leaf ethyl acetate; ICLHA: *Ixora coccinea* leaf hydroalcoholic; ICFPE: *Ixora coccinea* flower petroleum ether; ICFCE: *Ixora coccinea* flower chloroform extract; ICFEA: *Ixora coccinea* flower ethyl acetate; ICFHA: *Ixora coccinea* flower hydroalcoholic; IALPE: *Ixora alba* leaf petroleum ether; IALCE: *Ixora alba* leaf chloroform extract; IALEA: *Ixora alba* leaf ethyl acetate; IALHA: *Ixora alba* leaf hydroalcoholic; IAFPE: *Ixora alba* flower petroleum ether; IAFCE: *Ixora alba* flower chloroform extract; IAFEA: *Ixora alba* flower ethyl acetate; IAFHA: *Ixora alba* flower hydroalcoholic

Plant and parts used	Solvent used	Extract	Extractive value in %	Plant and parts used	% weight of the extract	Extract	Extractive value in %
*Ixora coccinea* leaf	Petroleum ether	ICLPE	2.65	*Ixora alba* leaf	Petroleum ether	IALPE	0.99
*Ixora coccinea* leaf	Chloroform	ICLCE	1.9	*Ixora alba* leaf	Chloroform	IALCE	3.39
*Ixora coccinea* leaf	Ethyl acetate	ICLEA	0.939	*Ixora alba* leaf	Ethyl acetate	IALEA	0.968
*Ixora coccinea *leaf	Hydroalcoholic	ICLHA	5.1	*Ixora alba* leaf	Hydroalcoholic	IALHA	2.8
*Ixora coccinea* flower	Petroleum ether	ICFPE	9.96	*Ixora alba* flower	Petroleum ether	IAFPE	1.16
*Ixora coccinea* flower	Chloroform	ICFCE	1.911	*Ixora alba* flower	Chloroform	IAFCE	1.143
*Ixora coccinea* flower	Ethyl acetate	ICFEA	0.9385	*Ixora alba* flower	Ethyl acetate	IAFEA	1.778
*Ixora coccinea* flower	Hydroalcoholic	ICFHA	3.7	*Ixora alba* flower	Hydroalcoholic	IAFHA	8.1

Elemental analysis

The contents of the elements detected in dried leaves and flowers are shown in Table 2.

**Table 2:** Elements detected and their content by elemental analysis BLQ: below limit of quantification; LOQ: limit of quantification

Plant used for the analysis	Arsenic (As) mg/kg	Cadmium (Cd) mg/kg	Lead (Pb) mg/kg	Mercury (Hg) mg/kg	Iron (Fe) mg/kg
*Ixora coccinea* leaf (ICL)	BLQ (LOQ-0.1)	BLQ (LOQ-0.1)	1.29	BLQ (LOQ-0.1)	42
*Ixora coccinea* flower (ICF)	BLQ (LOQ-0.1)	BLQ (LOQ-0.1)	1	BLQ (LOQ-0.1)	38
*Ixora alba* leaf (IAL)	BLQ (LOQ-0.1)	BLQ (LOQ-0.1)	0.40	BLQ (LOQ-0.1)	78
*Ixora alba* flower (IAF)	BLQ (LOQ-0.1)	BLQ (LOQ-0.1)	0.21	BLQ (LOQ-0.1)	104

Phytochemical analysis

The positive and negative results of phytochemical constituents present in the extract were shown as (+) and (-), respectively (Table 3).

**Table 3:** Photochemical analysis of petroleum ether, chloroform, ethyl acetate, and hydroalcoholic extracts of Ixora coccinea and Ixora alba leaves and flowers ICLPE: *Ixora coccinea* leaf petroleum ether; ICFPE: *Ixora coccinea* flower petroleum ether; IALPE: *Ixora alba *leaf petroleum ether; IAFPE: *Ixora alba* flower petroleum ether; ICLCE: *Ixora coccinea* leaf chloroform extract; ICFCE: *Ixora coccinea* flower chloroform extract; IALCE: *Ixora alba* leaf chloroform extract; IAFCE: *Ixora alba* flower chloroform extract; ICLEA: *Ixora coccinea* leaf ethyl acetate; ICFEA: *Ixora coccinea *flower ethyl acetate; IALEA: *Ixora alba* leaf ethyl acetate; IAFEA: *Ixora alba* flower ethyl acetate; ICLHA: *Ixora coccinea* leaf hydroalcoholic; ICFHA: *Ixora coccinea* flower hydroalcoholic; IALHA: *Ixora alba* leaf hydroalcoholic; IAFHA: *Ixora alba* flower hydroalcoholic; + indicates positive; − depicts negative

Phytoconstituents	Petroleum ether extract				Chloroform extract				Ethyl acetate extract				Hydroalcoholic extract				
	ICLPE	ICFPE	IALPE	IAFPE	ICLCE	ICFCE	IALCE	IAFCE	ICLEA	ICFEA	IALEA	IAFEA	ICLHA		ICFHA	IALHA	IAFHA
Alkaloid	−	−	−	−	+	+	+	+	+	+	+	+	−		−	−	−
Carbohydrate	−	−	−	−	+	+	+	+	−	−	−	−	−		−	−	−
Saponin	−	−	−	−	−	−	−	−	−	−	−	−	+		−	−	−
Glycosides	−	−	−	−	−	−	−	−	−	−	−	−	−		−	−	−
Phytosterols	+	+	+	+	−	+	−	+	+	+	−	+	+		+	+	+
Phenols	−	−	−	−	+	−	+	+	−	−	−	−	−		−	−	−
Tannins	−	−	−	−	−	+	−	+	−	−	−	−	−		−	−	−
Flavonoids	+	+	+	+	−	+	−	+	+	+	+	+	+		+	+	+
Proteins and amino acids	+	+	+	+	+	−	+	−	−	−	−	−	−		−	−	−
Diterpene	−	−	−	−	−	+	−	+	+	+	+	+	+		+	−	−

DPPH assay

**Figure 1:**
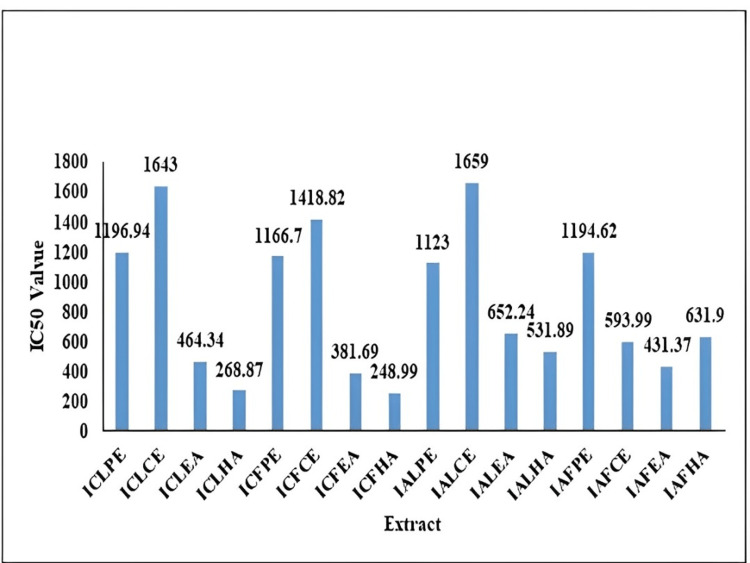
IC50 value of Ixora coccinea and Ixora alba leaf and flower ICLPE: *Ixora coccinea* leaf petroleum ether; ICLCE: *Ixora coccinea* leaf chloroform extract; ICLEA: *Ixora coccinea* leaf ethyl acetate; ICLHA: *Ixora* *coccinea* leaf hydroalcoholic; ICFPE: *Ixora coccinea* flower petroleum ether; ICFCE: *Ixora coccinea* flower chloroform extract; ICFEA: *Ixora coccinea* flower ethyl acetate; ICFHA: *Ixora coccinea* flower hydroalcoholic; IALPE: *Ixora alba* leaf petroleum ether; IALCE: *Ixora alba* leaf chloroform extract; IALEA: *Ixora alba *leaf ethyl acetate; IALHA: *Ixora alba* leaf hydroalcoholic; IAFPE: *Ixora alba* flower petroleum ether; IAFCE: *Ixora alba* flower chloroform extract; IAFEA: *Ixora alba* flower ethyl acetate; IAFHA: *Ixora alba* flower hydroalcoholic; IC50: half-maximal inhibitory concentration

The mean IC50 values of all extracts are reported (Table 4). A graph representation of the IC50 values of various extracts of *I. coccinea *and *I. alba *was shown (Figure 1).

**Table 4:** IC50 value of Ixora coccinea and Ixora alba leaf and flower ICLPE: *Ixora coccinea* leaf petroleum ether; ICLCE: *Ixora coccinea* leaf chloroform extract; ICLEA: *Ixora coccinea* leaf ethyl acetate; ICLHA: *Ixora coccinea* leaf hydroalcoholic; ICFPE: *Ixora coccinea* flower petroleum ether; ICFCE: *Ixora coccinea* flower chloroform extract; ICFEA: *Ixora coccinea* flower ethyl acetate; ICFHA: *Ixora coccinea *flower hydroalcoholic; IALPE: *Ixora alba* leaf petroleum ether; IALCE: *Ixora alba* leaf chloroform extract; IALEA: *Ixora alba* leaf ethyl acetate; IALHA: *Ixora alba* leaf hydroalcoholic; IAFPE: *Ixora alba* flower petroleum ether; IAFCE: *Ixora alba* flower chloroform extract; IAFEA: *Ixora alba* flower ethyl acetate); IAFHA: *Ixora alba* flower hydroalcoholic; SD: standard deviation); IC50: half-maximal inhibitory concentration Values are means of triplicate experiments

Sample ID	IC50 value mean ± SD	Extract	IC50 value mean ± SD
Ixora coccinea		Ixora alba	
ICLPE	1196.94 ± 0.04	IALPE	1123 ± 0.02
ICLCE	1643 ± 0.02	IALCE	1659 ± 0.01
ICLEA	464.34 ± 0.05	IALEA	652.24 ± 0.00
ICLHA	268.87 ± 0.01	IALHA	531.89 ± 0.01
ICFPE	1166.7 ± 0.01	IAFPE	1194.62 ± 0.01
ICFCE	1418.82 ± 0.02	IAFCE	593.99 ± 0.03
ICFEA	381.69 ± 0.04	IAFEA	431.37 ± 0.02
ICFHA	248.99 ± 0.05	IAFHA	631.9 ± 0.01

Total antioxidant assay

**Figure 2:**
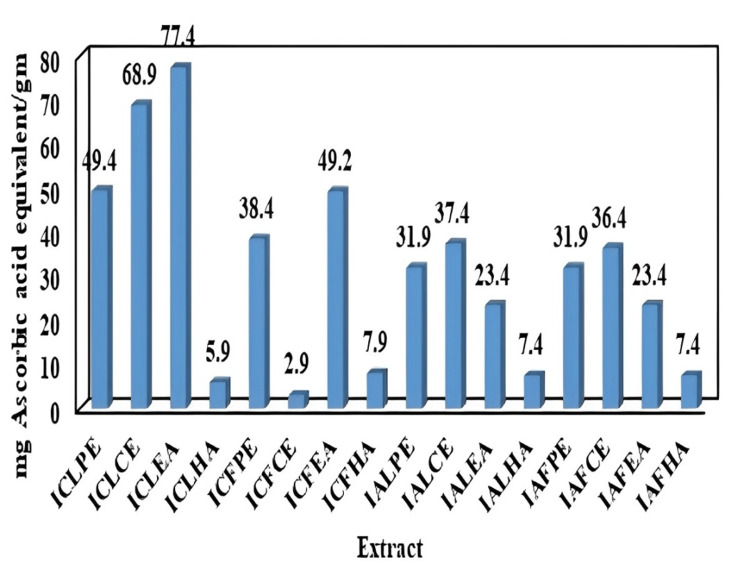
Milligram of AAE per gram of Ixora coccinea and Ixora alba leaf and flower ICLPE: *Ixora coccinea* leaf petroleum ether; ICLCE: *Ixora coccinea* leaf chloroform extract; ICLEA: *Ixora coccinea* leaf ethyl acetate; ICLHA: *Ixora coccinea* leaf hydroalcoholic; ICFPE: *Ixora coccinea* flower petroleum ether; ICFCE: *Ixora coccinea* flower chloroform extract; ICFEA: *Ixora coccinea* flower ethyl acetate; ICFHA: *Ixora coccinea* flower hydroalcoholic; IALPE: *Ixora alba* leaf petroleum ether; IALCE: *Ixora alba* leaf chloroform extract; IALEA: *Ixora alba* leaf ethyl acetate; IALHA: *Ixora alba* leaf hydroalcoholic; IAFPE: *Ixora alba* flower petroleum ether; IAFCE: *Ixora alba* flower chloroform extract; IAFEA: *Ixora alba* flower ethyl acetate; IAFHA: *Ixora alba* flower hydroalcoholic; AAE: ascorbic acid equivalent

The milligrams of AAE per gram of extract are reported (Table 5) and the graph represents the comparison of all the extracts (Figure 2).

**Table 5:** Milligrams of AAE per gram of Ixora coccinea and Ixora alba leaf and flower ICLPE: *Ixora coccinea* leaf petroleum ether; ICLCE: *Ixora coccinea* leaf chloroform extract; ICLEA: *Ixora coccinea* leaf ethyl acetate; ICLHA: *Ixora coccinea* leaf hydroalcoholic; ICFPE: *Ixora coccinea* flower petroleum ether; ICFCE: *Ixora coccinea* flower chloroform extract; ICFEA: *Ixora coccinea* flower ethyl acetate; ICFHA: *Ixora coccinea* flower hydroalcoholic; IALPE: *Ixora alba* leaf petroleum ether; IALCE: *Ixora alba* leaf chloroform extract; IALEA: *Ixora alba* leaf ethyl acetate; IALHA: *Ixora alba* leaf hydroalcoholic; IAFPE: *Ixora alba* flower petroleum ether; IAFCE: *Ixora alba* flower chloroform extract; IAFEA: *Ixora alba* flower ethyl acetate; IAFHA: *Ixora alba* flower hydroalcoholic; SD: standard deviation; AAE: ascorbic acid equivalents Values are means of triplicate experiments

Extract	mg AAE/g mean ± SD	Extract	mg AAE/g mean ± SD
Ixora coccinea		Ixora alba	
ICLPE	49.4 ± 0.01	IALPE	31.9 ± 0.03
ICLCE	68.9 ± 0.03	IALCE	37.4 ± 0.02
ICLEA	77.4 ± 0.05	IALEA	23.4 ± 0.01
ICLHA	5.9 ± 0.02	IALHA	7.4 ± 0.02
ICFPE	38.4 ± 0.01	IAFPE	31.9 ± 0.02
ICFCE	2.9 ± 0.04	IAFCE	36.4 ± 0.03
ICFEA	49.2 ± 0.03	IAFEA	23.4 ± 0.04
ICFHA	7.9 ± 0.05	IAFHA	7.4 ± 0.01

Antimicrobial studies

Extracts of* I. coccinea* and *I. alba* leaf and flower parts showed significant antimicrobial activity (Tables 6-7).

**Table 6:** Antimicrobial activity of petroleum ether, chloroform, ethyl acetate, and hydroalcoholic extracts of Ixora coccinea and Ixora alba leaf and flower ICLPE: *Ixora coccinea* leaf petroleum ether; ICLCE: *Ixora coccinea *leaf chloroform extract; ICLEA: *Ixora coccinea* leaf ethyl acetate; ICLHA: *Ixora coccinea* leaf hydroalcoholic; ICFPE: *Ixora coccinea* flower petroleum ether; ICFCE: *Ixora coccinea* flower chloroform extract; ICFEA: *Ixora coccinea* flower ethyl acetate; ICFHA: *Ixora coccinea* flower hydroalcoholic; IALPE: *Ixora alba* leaf petroleum ether; IALCE: *Ixora alba* leaf chloroform extract; IALEA: *Ixora alba* leaf ethyl acetate; IALHA: *Ixora alba* leaf hydroalcoholic; IAFPE: *Ixora alba* flower petroleum ether; IAFCE: *Ixora alba* flower chloroform extract; IAFEA: *Ixora alba* flower ethyl acetate; IAFHA: *Ixora alba* flower hydroalcoholic; STD: standard chloramphenicol; + depicts positive; - depicts negative

Microbial strains		Petroleum ether extract				Chloroform extract				Ethyl acetate extract				Hydroalcoholic extract				
	STD	ICLPE	ICFPE	IALPE	IAFPE	ICLCE	ICFCE	IALCE	IAFCE	ICLEA	ICFEA	IALEA	IAFEA	ICLHA		ICFHA	IALHA	IAFHA
Staphylococcus aureus	+	−	−	−	−	−	−	−	−	+	+	+	+	+		+	+	+
Streptococcus pyogenes	+	−	−	−	−	−	−	−	−	−	−	−	−	−		−	−	−
Enterococcus faecalis	+	−	−	−	−	−	−	−	−	+	+	+	+	+		+	+	+
Escherichia coli	+	−	−	−	−	−	−	−	−	−	−	−	−	−		−	−	−
Klebsiella pneumoniae	+	−	−	−	−	−	−	−	−	−	−	−	−	−		−	−	−
Pseudomonas aeruginosa	+	−	−	−	−	−	−	−	−	−	−	−	−	−		−	−	−
Candida albicans	+	−	−	−	−	−	−	−	−	+	+	+	+	+		+	+	+
Cryptococcus neoformans	+	−	−	−	−	−	−	−	−	−	−	−	−	−		−	−	−

**Table 7:** Zone of inhibition of Ixora coccinea and Ixora alba ICLEA: *Ixora coccinea* leaf ethyl acetate; ICFEA: *Ixora coccinea* flower ethyl acetate; IALEA: *Ixora alba* leaf ethyl acetate; IAFEA: *Ixora alba* flower ethyl acetate; ICLHA: *Ixora coccinea* leaf hydroalcoholic; ICFHA: *Ixora coccinea* flower hydroalcoholic; IALHA: *Ixora alba* leaf hydroalcoholic; IAFHA: *Ixora alba* flower hydroalcoholic; STD: chloramphenicol standard

	ICLEA				ICFEA				IALEA				IAFEA			
Sample	STD	250 µg	500 µg	1000 µg	STD	250 µg	500 µg	1000 µg	STD	250 µg	500 µg	1000 µg	STD	250 µg	500 µg	1000 µg
Organism		Zone of inhibition (mm)							Zone of inhibition (mm)							
Staphylococcus aureus	18	10	14	15	17	10	12	14	15	6	8	10	15	6	8	9
Enterococcus faecalis	15	8	10	12	16	10	12	13	16	8	10	11	17	12	14	15
Candida albicans	20	8	10	12	21	9	12	14	21	8	10	12	20	8	9	10
	ICLHA				ICFHA				IALHA				IAFHA			
Sample	STD	250 µg	500 µg	1000 µg	STD	250 µg	500 µg	1000 µg	STD	250 µg	500 µg	1000 µg	STD	250 µg	500 µg	1000 µg
Organism	Zone of inhibition (mm)								Zone of inhibition (mm)							
Staphylococcus aureus	15	8	10	12	13	10	12	13	18	10	12	14	20	10	12	14
Enterococcus faecalis	15	10	11	12	15	9	10	12	16	13	14	15	18	12	13	14
Candida albicans	20	10	12	13	20	9	10	11	21	13	15	17	18	11	12	14

MIC

**Figure 3:**
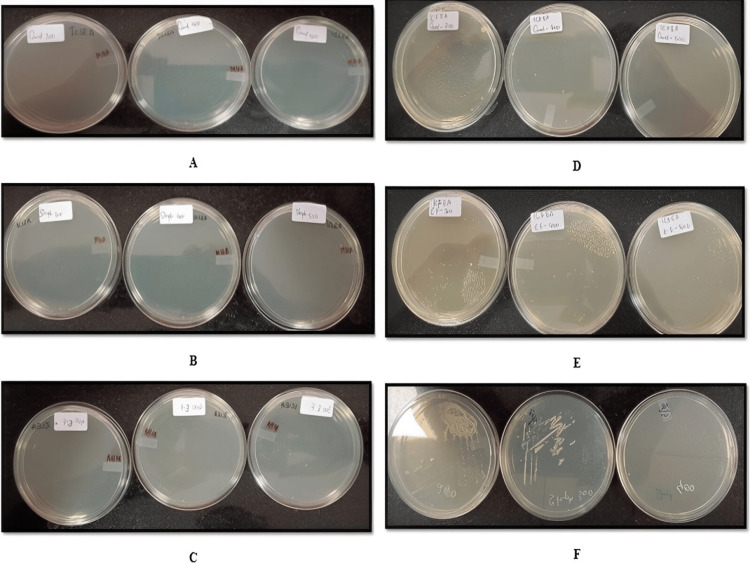
MIC of ethyl acetate extract of Ixora coccinea leaf and flower A: MIC of *Ixora coccinea* leaf ethyl acetate extract in *Candida albicans. *B: MIC of *Ixora coccinea* leaf ethyl acetate extract in *Staphylococcus aureus. *C: MIC of *Ixora coccinea* leaf ethyl acetate extract in *Enterococcus faecalis. *D: MIC of *Ixora coccinea* flower ethyl acetate extract in *Candida albicans. *E: MIC of *Ixora coccinea* flower ethyl acetate extract in *Enterococcus faecalis. *F: MIC of *Ixora coccinea* flower ethyl acetate extract in *Staphylococcus aureus* MIC: minimum inhibitory concentration

The MICs of the ethyl acetate extract of *Ixora coccinea *leaf and flower against *Staphylococcus aureus*, *Enterococcus faecalis*, and *Candida albicans* are reported (Figure 3). The MICs of the ethyl acetate extract and hydroalcoholic extracts of *I. coccinea* and *I. alba* against *Staphylococcus aureus*,*Enterococcus faecalis*, and *Candida albicans* are reported (Table 8).

**Table 8:** Minimal inhibitory concentrations of ethyl acetate and hydroalcoholic extracts of Ixora coccinea and Ixora alba leaf and flower ICLEA: *Ixora coccinea* leaf ethyl acetate; ICFEA: *Ixora coccinea* flower ethyl acetate; IALEA: *Ixora alba* leaf ethyl acetate; IAFEA: *Ixora alba* flower ethyl acetate; ICLHA: *Ixora coccinea *leaf hydroalcoholic; ICFHA: *Ixora coccinea* flower hydroalcoholic; IALHA: *Ixora alba* leaf hydroalcoholic; IAFHA: *Ixora alba* flower hydroalcoholic

Microbial strain	MIC of the plant extract							
	Ixora coccinea				Ixora alba			
	ICLEA	ICFEA	ICLHA	ICFHA	IALEA	IAFEA	IALHA	IAFHA
Candida albicans	500 µg	400 µg	400 µg	400 µg	400 µg	400 µg	300 µg	1000 µg
Enterococcus faecalis	400 µg	500 µg	300 µg	1000 µg	1000 µg	1000 µg	400 µg	1000 µg
Staphylococcus aureus	500 µg	500 µg	300 µg	1000 µg	1000 µg	1000 µg	400 µg	1000 µg

Total flavonoid content

**Figure 4:**
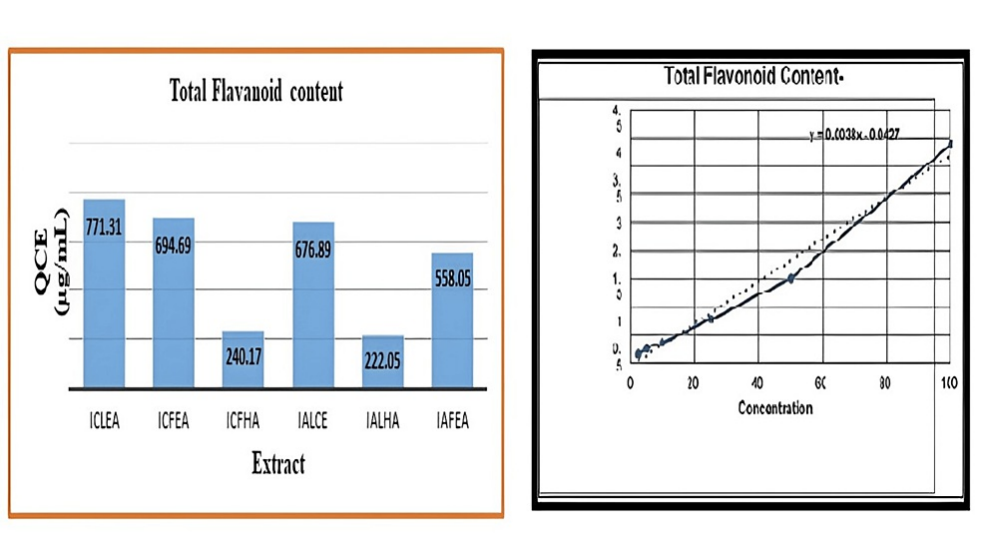
QCE (µg/mL) in Ixora coccinea and Ixora alba (leaf and flower) and the calibration curve of QCE (25-1000 µg/mL) ICLEA: *Ixora coccinea* leaf ethyl acetate; ICFEA: *Ixora coccinea *flower ethyl acetate; ICFHA: *Ixora coccinea* flower hydroalcoholic; IALCE: *Ixora alba* leaf chloroform extract; IALHA: *Ixora alba* leaf hydroalcoholic; IAFEA: *Ixora alba* flower ethyl acetate; QCE: quercetin

The results of the total flavonoid content of different extracts, *I. coccinea *and *I. alba*, are reported (Table 9, Figure 4).

**Table 9:** Total flavonoid content of QCE (µg/mL) ICLEA: *Ixora coccinea* leaf ethyl acetate; ICFEA: *Ixora coccinea* flower ethyl acetate; ICFHA: *Ixora coccinea *flower hydroalcoholic; IALCE: *Ixora alba* leaf chloroform extract; IALHA: *Ixora alba* leaf hydroalcoholic; IAFEA: *Ixora alba* flower ethyl acetate; QCE: quercetin; * mean of triplicate

Concentration of QCE (µg/mL)	*Absorbance of the standard at 415 nm	Sample	Concentration of the extract (mg/mL)	*Absorbance of the extract at 415 nm	Total flavonoid content (QCE) (µg/mL)
25	0.1587	ICLEA	10	2.8883	771.31
50	0.2620	ICFEA	10	2.5972	694.69
100	0.3721	ICFHA	10	0.8699	240.17
250	0.7797	IALCE	10	2.5295	676.89
500	1.5032	IALHA	10	0.8011	222.05
1000	3.8906	IAFEA	10	2.0779	558.05

HPTLC analysis

**Figure 5:**
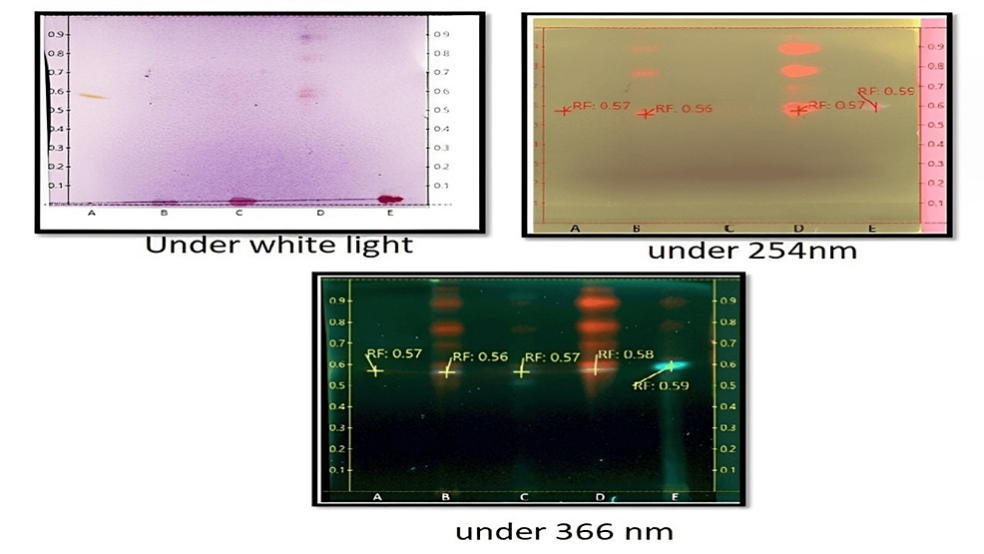
Visualization of a spot A: QCE; B: ICLEA; C: ICFEA; D: IALCE; E: IAFEA ICLEA: *Ixora coccinea* leaf ethyl acetate; ICFEA: *Ixora coccinea* flower ethyl acetate; IALCE: *Ixora alba* leaf chloroform extract; IAFEA: *Ixora alba* flower ethyl acetate; QCE: quercetin

The spots of the different extracts under different conditions are shown (Figure 5). The retention factor calculated for each extracted sample and QCE concentration were reported (Tables 10-11).

**Table 10:** Rf value A: QCE; B: ICLEA; C: ICFEA; D: IALCE; E: IAFEA ICLEA: *Ixora coccinea* leaf ethyl acetate; ICFEA: *Ixora coccinea* flower ethyl acetate; IALCE: *Ixora alba* leaf chloroform extract; IAFEA: *Ixora alba flower* ethyl acetate; Rf: retention factor; QCE: quercetin

Sample code	Sample name	Solvent system	Rf value under white light	Rf value under 254 nm	Rf value under 366 nm
A	QCE	Toluene:ethyl acetate:formic acid:methanol (5.5:3:1:0.5, v/v/v/v)	0.57	0.57	0.57
B	ICLEA		-	0.56	0.56
C	ICFEA		-	-	0.57
D	IALCE		0.58	0.57	0.58
E	IAFEA		-	0.59	0.59

**Table 11:** QCE concentration (µg/mL) in ICLEA and IALCE ICLEA: *Ixora coccinea* leaf ethyl acetate; IALCE: *Ixora alba* leaf chloroform extract; QCE: quercetin

Sample	QCE concentration (µg/mL)
ICLEA	24.44
IALCE	22.37

In vitro anticancer evaluation by MTT assay

**Figure 6:**
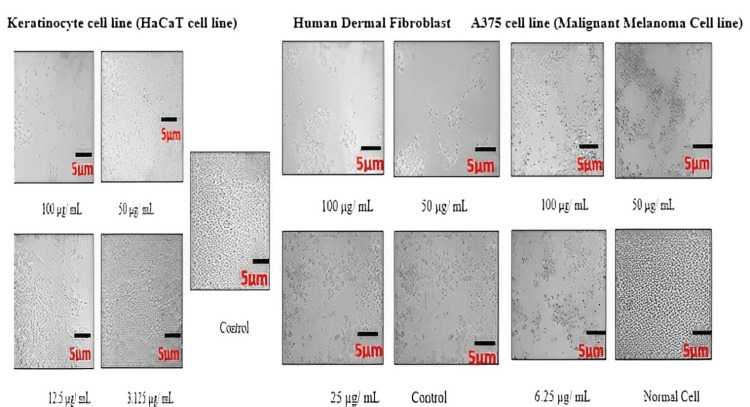
Visual live cells from different cell lines after I. coccinea leaf ethyl acetate extract treatment

**Figure 7:**
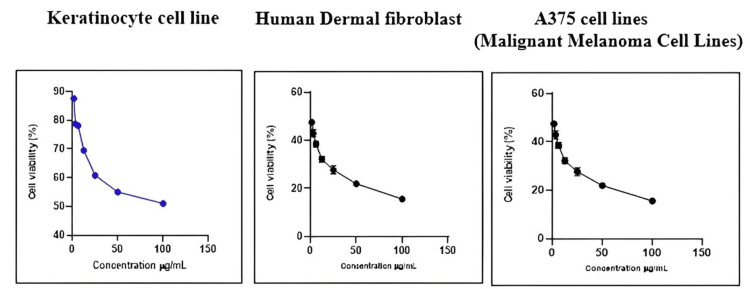
Percentage of cell viability of different cell lines after I. coccinea leaf ethyl acetate extract treatment

**Figure 8:**
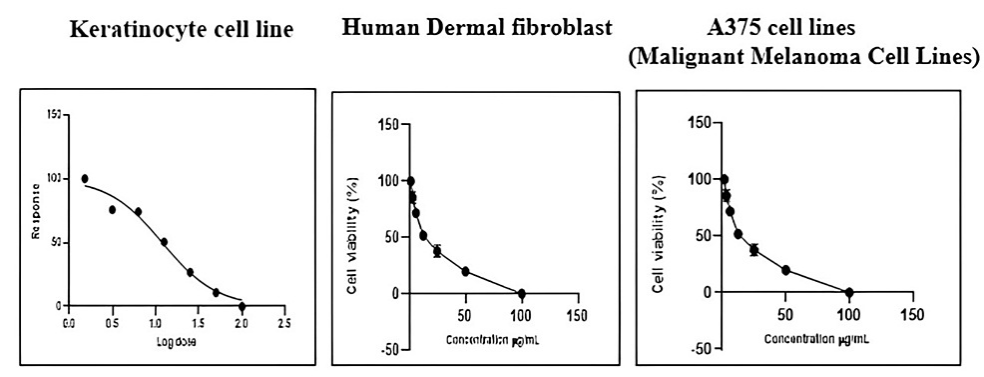
Nonlinear regression curve fit of different cell lines after I. coccinea leaf ethyl acetate extract treatment

**Figure 9:**
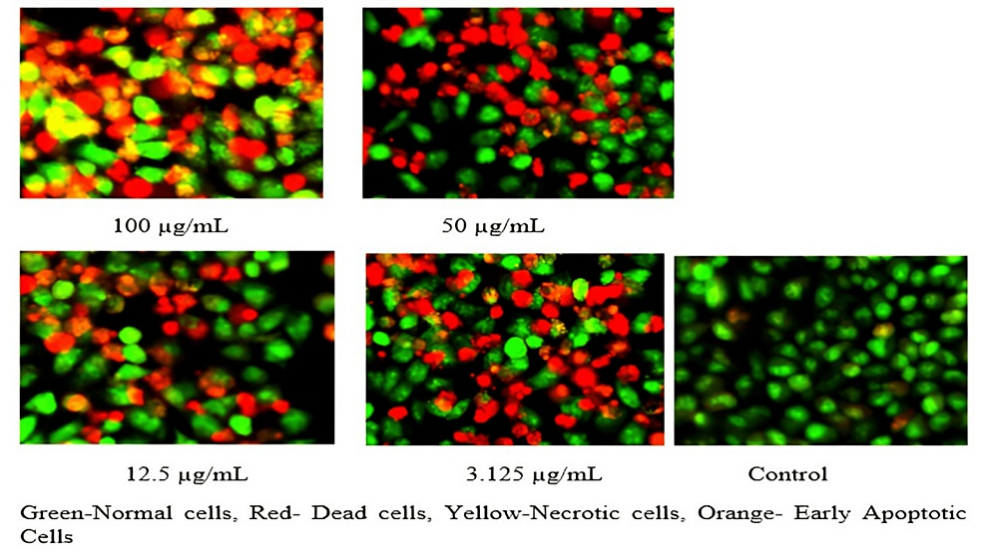
Live and dead staining with acridine orange and ethidium bromide in I. coccinea leaf ethyl acetate extract at different concentrations

The percentage of live and dead cells of the HaCaT cell line, A375 cell lines (malignant melanoma cell lines), and human dermal fibroblasts treated with ICLEA extract are reported (Table 12). An increase in the concentration of the ICLEA extract inhibited cell growth (Figure 6). The results for cell viability and the nonlinear regression curve fit are shown in Figures 7-8. Live and dead cells were differentiated by staining with acridine orange and ethidium bromide (Figure 9).

**Table 12:** Percentage of live and dead cells from different cell lines treated with I. coccinea leaf ethyl acetate extract SD: standard deviation; IC50: half-maximal inhibitory concentration; SEM: standard error of mean; HaCaT cell line: keratinocyte cell line Values are means of triplicate experiments

Cell line used	HaCaT cell line				Human dermal fibroblasts				A375 cell lines (malignant melanoma cell lines)			
Concentration of extract (µg/mL)	% cell death mean	SD	SEM	% live cells	% cell death	SD	SEM	% live cells	% cell death	SD	SEM	% live cells
100	48.98	1.39	0.80	51.02	63.98	0.56	0.32	36.02	86.30	7.95	4.59	13.70
50	44.97	0.98	0.56	55.03	49.77	2.17	1.25	50.23	84.24	1.27	0.73	15.76
25	39.18	1.74	1.00	60.82	41.19	0.66	0.38	58.81	66.34	7.29	4.21	33.66
12.5	30.48	1.42	0.82	69.52	32.58	0.58	0.34	67.42	59.98	5.94	3.43	40.02
6.25	21.84	1.27	0.74	78.16	29.97	0.56	0.32	70.03	53.55	4.69	2.71	46.45
3.125	21.22	1.01	0.58	78.78	27.63	0.65	0.38	72.37	37.54	2.40	1.39	62.46
1.5	12.38	1.58	0.91	87.62	15.42	4.12	2.38	84.58	24.67	3.57	2.06	75.33
Control				100				100.00				100
IC_50_				98.52 µg/mL	IC_50_			58.52 µg/mL	IC_50_			7.96 µg/mL

## Discussion

The selected* Ixora *species of *I. coccinea* and *I. alba* leaf and flowers were subjected to extraction by petroleum ether, chloroform, ethyl acetate, and hydroalcoholic (ethanol: water, 70:30, v/v) via the maceration method. The prepared extracts of *I. coccinea* were given the following IDs: ICLPE, ICLCE, ICLEAE, ICLHAE, ICFPE, ICFCE, ICFEAE, and ICFHAE. In the leaf part of the selected *Ixora* species, the highest percentage yield was obtained for the ICLHA extract. The prepared extracts of* I. alba *were given the following IDs: IALPE, IALCE, IALEAE, IALHAE, IAFPE, IACFCE, IAFEAE, and IAFHAE. In the flower of the selected *Ixora* species, the highest percentage yield was obtained for the ICFPE extract.

Elemental analysis performed by ICP-MS proved that arsenic (As), cadmium (Cd), and mercury (Hg) levels were below the limit of quantification. Lead (Pb) was detected to be very low, and iron (Fe) content was present in the dried leaves and flowers of *I. coccinea* and *I. alba*.

All extracts were tested for the presence of phytoconstituents using standard procedures. In phytochemical analysis, alkaloids, phytosterols, flavonoids, proteins, amino acids, and diterpenes were predominantly present in chloroform, ethyl acetate, and hydroalcoholic extracts of *I. coccinea* and *I. alba*.

In the DPPH assay, the percentage of the scavenging effect of the plant extract was estimated. The IC50 value was calculated. The hydroalcoholic extract of *I. coccinea* was shown to have a good scavenging effect compared with other extracts. Less inhibition was reported in the petroleum ether extract. The total antioxidant assay was performed using the phosphomolybdenum method. The highest total antioxidant capacity was proven in ICLEA at 77.4 ± 0.05 mg; in ICFEA it was 49.2 ± 0.03 mg; in IALCE it was 37.4 ± 0.02 mg; and in IAFCE it was 36.4 ± 0.03 mg.

The antimicrobial activity of *I. coccinea*, *I. alba* leaf, and flower extracts was evaluated against various bacterial and fungal strains such as *Staphylococcus aureus*, *Streptococcus pyogenes*, *Enterococcus faecalis*,* Escherichia coli*, *Klebsiella pneumoniae*, *Pseudomonas aeruginosa*, *Candida albicans*, and *Cryptococcus neoformans*. Petroleum ether and chloroform extract did not possess any zones of inhibition for the above eight strains. Ethyl acetate and hydroalcoholic extract showed a significant zone of inhibition with *Staphylococcus aureus*, *Enterococcus faecalis*, and *Candida albicans*, which was comparable with standard chloramphenicol. Ethyl acetate and hydroalcoholic extract showed antimicrobial activity, which may be due to the presence of phytosterol and flavonoid phytoconstituents in *I. coccinea* and *I. alba*. Results concluded that all ethyl acetate and hydroalcoholic extract fractions showed ≤400 µg of antimicrobial activity with all three strains.

Using the aluminum chloride method, the total flavonoid content was determined. In four extracts (ICLEA, ICFEA, IALCE, and IAFEA), the QCE content was greater than 500 µg/mL, and two extracts (ICFHA and IALHA) had a lower total flavonoid content. From the results, it was evident that a predominant QCE appearance was seen for both ICLEA and IALCE under all three illumination sources and was concluded by the retention factor; in particular, IALCE has bright red/yellow spots under white light similar to QCE. Only at 366 nm was the fluorescence QCE appearance observed for ICFEA. At 366 nm, fluorescence was observed for sample IAFEA, hinting that a flavone-type flavonoid may be present. Among the four samples, IAFEA displayed a single band under different illumination sources, indicating that it is a highly pure compound compared with the other extracts. The ICLEA extract has a high flavonoid content; the band produced was equivalent to QCE; therefore, it is identified as a promising extract for phytosome formulation.

It was found that 100 µg of ICLEA extract showed 48.98% inhibition in the HaCaT cell line. The IC50 value was found to be 98.52 µg/mL. Treatment of the ICLEA extract sample in the HaCaT cell line and human dermal fibroblasts did not affect normal cell functioning. It was proven that ICLEA did not cause any damage to normal cells. The ICLEA extract treatment on A375 cell lines (malignant melanoma cell lines) at various concentrations proved that an increase in the concentration increases cell growth inhibition.

Pornima et al. reported that ethanol, water, and hydroethanolic extracts of *I. coccinea* leaves have antioxidant potential [22]. Haji et al., by HPLC analysis, identified that pyrocatechol, catechin, and chlorogenic acid were most abundant in the root of *I. coccinea*. They proved that methanol has antioxidant and antibacterial effects [23].

Panikkar et al. formulated an ayurvedic oil from the *I. coccinea* and *Cortus sativum* flowers. The formulation was subjected to animals to identify the prevention of Dalton's lymphoma, a solid tumor, and the oil was reported to arrest the further growth of the tumor cell [24].

Latha et al. reported that the extract of *I. coccinea* flower obtained from n-Hexane, which was administered to Swiss albino mice for skin carcinogenic studies, caused a significant decrease in the mean number of papillomas in these mice. By spectrometrically identifying the presence of triterpenoid ursolic acid, which has already been reported to significantly prevent skin tumors [25].

Prabhu et al. evaluated the in vitro cytotoxicity activity of a methanolic extract of fresh flowers from *I. coccinea*. GC-MS analysis of the methanol extract identified the presence of cyclopentaneundecanoic acid, methyl ester (40.36%), and dibutyl phthalate (26.83%) as the major compounds. The methanolic extract showed an IC50 value of 250 µg/mL and 300 µg/mL against DLA and EAC cell lines, respectively [26].

Limitations

The phytosome gel formulation from the identified active fraction of the *I. coccinea* leaf ethyl acetate extract has not been prepared. Thus, phytosome preparation and evaluation of gel properties shall be studied. In the current study, the in vitro cytotoxicity effect in a cell line was only proved; further phytosomal gel in vivo studies for acute dermal toxicity and cell-induced cytotoxicity effects can be conducted to support the skin cancer activity of the *I. coccinea* leaf active fraction.

## Conclusions

From this preliminary analysis, it was concluded that *I. coccinea* leaf ethyl acetate has promising antioxidant and antimicrobial properties. This may be due to the presence of a high flavonoid content. The in vitro cytotoxicity by the MTT assay has proven that this extract does not affect normal cells; it inhibits only A375 cell lines (malignant melanoma cell lines) at a low concentration. Various studies have shown that plant extracts containing flavonoids can be expressed as phytosomes to reduce problems arising from pharmacokinetics and bioavailability. Hence, phytosomes can be formulated from *I. coccinea *leaf ethyl acetate extract, which may be a potent candidate for various ailments.
